# Connecting Metabolic Pathways: Sigma Factors in *Streptomyces* spp.

**DOI:** 10.3389/fmicb.2017.02546

**Published:** 2017-12-19

**Authors:** Di Sun, Cong Liu, Jingrong Zhu, Weijie Liu

**Affiliations:** School of Life Sciences, Jiangsu Normal University, Xuzhou, China

**Keywords:** *Streptomyces*, σ factor, primary metabolism, secondary metabolism, morphological differentiation, stress response

## Abstract

The gram-positive filamentous bacterium *Streptomyces* is one of the largest resources for bioactive metabolites, particularly antibiotics. Antibiotic production and other metabolic processes are tightly regulated at the transcriptional level. Sigma (σ) factors are components of bacterial RNA polymerases that determine promoter specificity. In *Streptomyces*, σ factors also play essential roles in signal transduction and in regulatory networks, thereby assisting in their survival in complex environments. However, our current understanding of σ factors in *Streptomyces* is still limited. In this mini-review, we demonstrate the roles of *Streptomyces* σ factors, illustrating that these serve as linkers of different metabolic pathways. Further investigations on σ factors may improve our knowledge of *Streptomyces* physiology and benefit exploitation of *Streptomyces* resources.

## Introduction

*Streptomyces* is the largest genus of Actinobacteria and is characterized by complex morphological differentiation and the ability to produce valuable secondary metabolites (reviewed by Demain, 2002; Challis and Hopwood, 2003; Flardh and Buttner, 2009). This genus possesses complex regulatory systems that coordinate various biological processes and allows survival in their habitats. Transcriptional regulation is one of the most extensively studied regulatory mechanisms, with RNA polymerase (RNAP) playing a central role in transcription. Transcription initiation requires a holo-RNAP complex, which is composed of a core-RNAP (α2ββ′ω) and a dissociable sigma (σ) factor that recognizes specific promoter elements and facilitates transcription initiation (reviewed by Browning and Busby, 2004; Paget, 2015). Although the importance of σ factors has been established, further investigations on its regulatory mechanisms are warranted (reviewed by Feklistov et al., 2014; Browning and Busby, 2016). Sigma factors are not only a distinct subunit of RNAPs, but also an essential part of signal transduction systems. Endogenous and exogenous signals regulate the expression and activity of σ factors, thereby modifying the transcriptome (Cho et al., 2001; Takano et al., 2005; Hesketh et al., 2007; Feng et al., 2011). Moreover, various σ factors act as master regulators, regulating more than one biological process (Lee et al., 2005; Kim et al., 2012). In this mini-review, we provide an overview of the functions of σ factors in the life cycle of *Streptomyces* and illustrate that these also link different metabolic pathways, which benefits further investigation of σ factors and exploitation of *Streptomyces* resources.

## Classification of σ Factors

**FIGURE 1:**
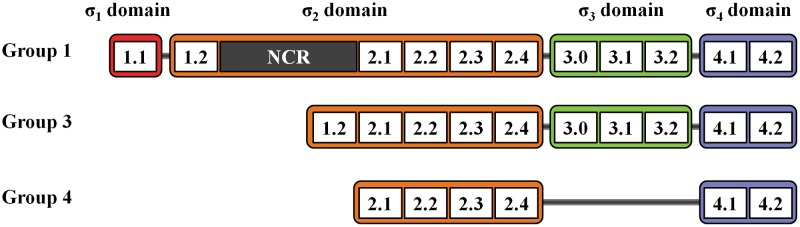
σ factor domains in the σ^70^ family. Domain organization of σ^70^ family members from groups 1, 3, and 4.

Bacterial σ factors can be divided into two families, namely, σ^70^ and σ^54^. The σ^54^ family, which contains only one member that is distinct from σ^70^ proteins, has been excluded from this review. The σ^70^ family is named after the *Escherichia coli* housekeeping σ^70^ and consists of four subgroups (1, 2, 3, and 4) based on differential physical functions, phylogenic relationships, and possession of conserved domains σ_1_ (region 1.1), σ_2_ (region 1.2, 2.1–2.4), σ_3_ (region 3.0–3.2), and σ_4_ (region 4.1–4.2) (**Figure 1**) (reviewed by Paget, 2015). The number of members in the σ^70^ family, particularly those in groups 3 and 4, is highly variable among bacterial species. *Streptomyces* spp. encodes various σ factors to survive in complex environments and coordinate complicated physiological processes. This mini-review focuses on the *Streptomyces* σ^70^ family proteins.

## Group 1 σ Factors

Group 1 σ factors, including *E. coli* σ^70^ and its orthologs, possess all four σ domains and a non-conserved region (NCR) of variable length located between regions 1.2 and 2.1 (**Figure 1**). NCR is involved in core interactions and promoter escape (Leibman and Hochschild, 2007). Group 1 σ factors are responsible for the transcription of essential genes and are thus also called “housekeeping σ factors” (reviewed by Paget, 2015). In *Streptomyces*, principal housekeeping σ factor (σ^HrdB^) is not only required for essential gene expression, but is also involved in morphological differentiation and secondary metabolism (Fujii et al., 1996). Studies have revealed that σ^HrdB^ affects secondary metabolism in at least two ways. First, σ^HrdB^ initiates the transcription of pathway-specific regulatory genes that are essential to secondary metabolite biosynthesis. For example, in *S. coelicolor*, σ^HrdB^ regulates the biosynthesis of actinohordin (ACT) and undecylprodigiosin (RED) by initiating the transcription of the pathway-specific regulatory genes *actII*-ORF4 and *redD* (Fujii et al., 1996). Similarly, in *S. avermitilis*, σ^HrdB^ initiates the transcription of the pathway-specific regulatory gene *aveR* to promote the biosynthesis of avermectin (Zhuo et al., 2010). Moreover, the biosynthesis of secondary metabolites requires precursors and energy, which is produced by σ^HrdB^-dependent primary metabolism. Thus, σ^HrdB^ mediates the metabolic flux from primary to secondary metabolism.

Because σ^HrdB^ connects primary metabolism with secondary metabolism, genetic manipulation of the *hrdB* gene has been utilized as a strategy in enhancing antibiotic production. The engineering of *hrdB* can be performed by at least two methods. In the first method, the site-mutated *hrdB* gene that triggers the upregulation of *aveR* transcription is introduced into avermectin high-producing strain 3–115, thereby improving avermectin B1a yield by over 50% (Zhuo et al., 2010). In the second method, as the strength of the *hrdB* promoter (*hrdBp*) is greater than other commonly used promoters in *Streptomyces*, the native promoters of the antibiotic biosynthesis genes or pathway-specific regulatory genes are replaced with *hrdBp* to improve antibiotic yield. For example, nikkomycin yield is significantly enhanced by introducing *hrdBp*-driven *sanG*, which encodes the pathway-specific activator of nikkomycin biosynthesis in *S. ansochromogenes* TH322 (Du et al., 2013).

## Group 2 σ Factors

The structure of group 2 σ factors is highly similar to that of group 1 σ factors, except for the absence of region 1.1. Furthermore, group 2 σ factors are not essential to bacterial growth. The number of group 2 σ factors encoded by bacterial genomes is highly variable (**Table 1**) (Buttner and Lewis, 1992; Osanai et al., 2008; Battesti et al., 2011). The *Streptomyces* genome usually contains three group 2 σ factors: σ^HrdA^, σ^HrdC^, and σ^HrdD^ (Buttner et al., 1990; Forsman and Granstrom, 1992; Kormanec et al., 1992; Wang et al., 2015). The majority of studies have focused on σ^HrdD^.

**Table 1:** Number of σ factors in actinobacteria species.

Strains	Group 1	Group 2	Group 3	Group 4	Total
*Streptomyces avermitilis* ATCC31267	1	2	10	47	60
*Streptomyces coelicolor* A3(2)	1	3	10	50	64
*Streptomyces griseus* IFO13350	1	2	10	39	52
*Streptomyces scabies* 87.22	1	2	10	48	61
*Streptomyces venezuelae* ATCC10712	1	2	9	28	40
*Micromonospora aurantiaca* ATCC 27029	1	3	3	31	38
*Mycobacterium tuberculosis* H37Rv	1	1	1	10	13
*Mycobacterium smegmatis* MC^2^ 155	1	1	1	21	24
*Corynebacterium glutamicum* ATCC13032	1	0	0	5	6


Although σ^HrdD^ is transcribed during growth, and the σ^HrdD^-RNAP holoenzyme can initiate transcription of genes that are involved in antibiotic biosynthesis (e.g., *actII*-ORF4, *redD*) and differentiation (e.g., *whiB*) *in vitro*, *hrdD* mutants do not exhibit changes in secondary metabolism and differentiation (Fujii et al., 1996; Kang et al., 1997), thereby prompting us to examine the biological significance of σ^HrdD^. Earlier studies have determined that *hrdD* can be induced by stresses such as envelope stress, oxidative damage, and hyperosmolarity (Kang et al., 1997; Paget et al., 2001; Lee et al., 2005), suggesting that σ^HrdD^ may be involved in stress response processes. Moreover, in pathogenic actinobacterium, *Mycobacterium tuberculosis*, group 2 σ factor σ_Mtb_^B^ can specifically bind to RbpA, a transcriptional activator without DNA-binding activity, and the activity of σ_Mtb_^B^ is stringently dependent on RbpA (Hu et al., 2014). These findings suggest that in *Streptomyces*, σ^HrdD^ may bind to an RbpA-like protein to form a complex, which is required for survival under stress conditions.

Although the expression of group 2 σ factors in *Streptomyces* may be related to multiple biological processes, knowledge of their biological significance remains limited. *E. coli* σ^S^, the most extensively studied group 2 σ factor, can affect the expression of about 500 target genes under stress conditions. It is not only involved in general stress responses but also in survival in the stationary phase (Weber et al., 2005; Battesti et al., 2011). Therefore, further investigations are warranted to clarify the underlying regulatory mechanisms of *Streptomyces* group 2 σ factors.

## Group 3 σ Factors

Group 3 σ factors possess σ_2_, σ_3_, and σ_4_ domains (**Figure 1**), which are referred to as alternative σ factors that are used to direct RNAP to genes involved in adaptive responses (reviewed by Gruber and Gross, 2003). In *Streptomyces*, alternative σ factors modulate multiple metabolic pathways in response to intracellular or extracellular signals (Kormanec and Sevcikova, 2002; Viollier et al., 2003; Dalton et al., 2007; Bobek et al., 2014).

σ^WhiG^, an alternative σ factor that regulates differentiation, orchestrates the formation of spores by activating the transcription of three regulatory genes, namely, *whiA*, *whiH*, and *whiI*, which are essential for cell division and spore maturation (Ryding et al., 1998; Ainsa et al., 1999; Kaiser and Stoddard, 2011). In *S. chattanoogensis*, σ^WhiG^ also regulates secondary metabolism. σ^WhiG^ promotes natamycin biosynthesis by directly activating the transcription of two natamycin biosynthetic genes, *scnC* (encodes an aminotransferase) and *scnD* (encodes a P450 monooxygenase). The deletion of *scnC* or *scnD* results in the accumulation of two natamycin analogs, 4,5-deepoxy-natamycinolide and 4,5-deepoxy-natamycin, respectively, but abolishes natamycin biosynthesis (Liu et al., 2015a,b). Besides, *whiG* expression is regulated by bis-(3′-5′)-cyclic dimeric GMP (c-di-GMP), an important second messenger that imparts global effects in *Streptomyces* (reviewed by Bush et al., 2015). A transcriptional repressor BldD negatively regulates *whiG* transcription in a (c-di-GMP)-dependent manner. At the vegetative growth stage, high concentrations of c-di-GMP lead to the formation of BldD-(c-di-GMP) complexes that bind to *whiG* promoter regions, repressing its transcription. When morphological differentiation starts, c-di-GMP concentrations decline, causing the release of BldD from the target DNA, thereby relieving transcriptional suppression of *whiG* (Elliot et al., 2001; Tschowri et al., 2014). Therefore, σ^WhiG^ responds to the intracellular signal molecule and coordinates morphological differentiation as well as secondary metabolism.

Except for σ^WhiG^, *S. coelicolor* has nine other alternative σ factors (σ^B^, σ^F^, σ^G^, σ^H^, σ^I^, σ^K^, σ^L^, σ^M^, and σ^N^), all belonging to the σ^B^-like σ factors, which are homologs of *Bacillus subtilis* σ^B^ (Cho et al., 2001; Lee et al., 2004). These σ^B^-like σ factors play important roles in the *Streptomyces* regulatory system (Kelemen et al., 1996; Kormanec and Sevcikova, 2002; Lee et al., 2005; Mao et al., 2009; Wang et al., 2010). Among the σ^B^-like σ factors, σ^B^ has been most extensively investigated. σ^B^ can control morphological differentiation and secondary metabolism by directly regulating differentiation-related genes (e.g., *dpsA* required for spore maturation and *whiB* that is required for aerial hyphae formation) and antibiotic biosynthetic genes (e.g., *redH* that encodes a phosphoenolpyruvate-utilizing enzyme and *redZ* that encodes a pathway-specific regulator for RED) (Lee et al., 2005; Facey et al., 2009, 2011). Furthermore, σ^B^ controls morphological differentiation and secondary metabolism by regulating the ppGpp synthesis (Lee et al., 2004). Guanosine tetraphosphate (ppGpp) is an extensively studied signaling nucleotide that is synthesized in nutrient-limited conditions. It interacts directly with RNAP to modulate the transcriptome (reviewed by Hauryliuk et al., 2015). In *S. coelicolor*, ppGpp affects differentiation and secondary metabolism by modulating the expression of genes required for aerial hyphae formation and pathway-specific regulators (Hesketh et al., 2007). σ^B^ influences intracellular ppGpp concentrations by directly regulating the ppGpp synthetase gene *relA* (Lee et al., 2004).

In addition, σ^B^ responds to environmental stress signals and activates the stress-related regulon. First, σ^B^ controls osmotic defensive responses by initiating the transcription of genes that are involved in the synthesis and uptake of osmotic compatible solutes (Lee et al., 2005; Fernandez-Martinez et al., 2009), as well as activating two other alternative σ factors that also contribute to osmotic stress responses, namely, σ^L^ and σ^M^, in a hierarchical order to strengthen the regulatory network (Lee et al., 2005). Second, σ^B^ is involved in oxidative stress responses by promoting cysteine and mycothiol (streptomycetes major thiol buffer) biosynthesis and the expression of catalase (Lee et al., 2005). Finally, the σ^B^ regulon includes cold shock proteins, thereby suggesting that it may be involved in cold shock responses (Lee et al., 2005). Interestingly, upon osmotic stress, σ^B^ rapidly activates the osmotic defense system and subsequently promotes the transcription of oxidative stress-related genes (Lee et al., 2005), suggesting that σ^B^ protects *S. coelicolor* from subsequent oxidative damage after osmotic shock. These findings indicate that σ^B^ governs multiple stress responses in *Streptomyces*. In summary, σ^B^ governs multiple biological processes and acts as a key element in the stress response system, thereby eliciting rapid responses to changes in habitat.

## Group 4 σ Factors

Group 4 σ factors are the most divergent members of the σ^70^ family and exclusively contain the σ_2_ (which lacks region 1.2) and σ_4_ domains (**Figure 1**). As group 4 σ factors usually correlate with extracytoplasmic stimuli, these are also called extracytoplasmic function (ECF) σ factors (reviewed by Staron et al., 2009; Paget, 2015). The *Streptomyces* genome encodes various ECF σ factors, which reflects their complex habitats and complicated metabolic processes (**Table 1**).

Extracytoplasmic function σ factors play essential roles in the stress response system of *Streptomyces* spp. One of the most extensively studied stress-responsive ECF σ factor is *S. coelicolor* σ^R^. It is induced by oxidants and translation-inhibiting antibiotics and subsequently activates the oxidative damage reparatory system, ribosome-associated products, and transcriptional regulators (e.g., RbpA) (Paget et al., 2001; Yoo et al., 2016; Lee et al., 2017). Additionally, during thiol-oxidative stress, σ^R^ is required to maintain the level and activity of σ^HrdB^, thereby suggesting a crosstalk between ECF σ factor and housekeeping σ factor during stress (Kim et al., 2012). These findings reveal that σ^R^ is a master regulator of defense responses against multiple stresses. On the other hand, σ^R^ is regulated at multiple levels. First, σ^R^ activity is regulated by its cognate anti-σ factor, RsrA. Under normal conditions, RsrA binds to σ^R^ and sequesters σ^R^ from RNAP. Under stress, RsrA undergoes structural changes that cause the release of σ^R^, which subsequently regulates its regulons (Kang et al., 1999). The expression of σ^R^ is also regulated at the transcriptional (promoted by transcriptional activator WblC), translational (repressed by translation initiation factor IF3), and post-translational (degraded by Clp proteases) levels (Kim et al., 2009; Yoo et al., 2016; Feeney et al., 2017). Taken together, σ^R^ is a pivotal element in the complex regulatory network of *Streptomyces* spp.

Extracytoplasmic function σ factors are involved in morphological differentiation. The widely distributed ECF σ factor, σ^BldN^, is required for aerial mycelium formation (Bibb et al., 2000). Studies have revealed that σ^BldN^ is responsible for the expression of differentiation-related proteins chaplin, rodlin, and BldM (Bibb et al., 2000, 2012). Deletion of *bldN* abolishes aerial hyphae formation, thereby causing the “bald” phenotype (Bibb et al., 2000; Yague et al., 2014).

Regulation of secondary metabolism by ECF σ factors is commonly identified among actinomycetes, and some pathway-specific ECF σ factors are located within the antibiotic biosynthesis gene cluster. σ^MibX^ in *Microbispora corallina* is the most extensively studied cluster-situated ECF σ factor that controls the biosynthesis and maturation of microbisporicin (Foulston and Bibb, 2010). Microbisporicin is synthesized by the *mib* gene cluster, which is composed of six transcriptional units (i.e., *mibJYZO*, *mibQ*, *mibR*, *mibXW*, *mibABCDTUV*, and *mibEFHSN*). The LuxR-family regulator MibR is responsible for the activation of *mibABCDTUV*, and the ECF σ factor σ^MibX^ initiates the transcription of *mibJYZO*, *mibQ*, *mibR*, *mibXW*, and *mibEFHSN*. Under normal conditions, the transcriptional activity of σ^MibX^ is sequestered by its cognate anti-σ factor MibW, abolishing microbisporicin production. Upon nutrient limitation, cellular ppGpp concentrations increase and subsequently activate *mibR* transcription in a σ^MibX^-independent manner, followed by the expression of the *mibABCDTUV* operon, thereby inducing the biosynthesis of immature microbisporicin. Subsequently, the immature antibiotic inactivates MibW, leading to the release of σ^MibX^, which in turn upregulates the expression of mature microbisporicin (Foulston and Bibb, 2011; Fernandez-Martinez et al., 2015). This model illustrates that antibiotics can function as a signal and an ECF-σ factor-(anti-σ factor) complex may play a crucial role in the signaling pathway.

In *Streptomyces*, the only identified cluster-situated ECF σ factor is σ^AntA^, which has been reported in *S. albus*. Unlike σ^MibX^, no anti-σ factor regulating the transcriptional activity of σ^AntA^ has been reported to date (Seipke et al., 2014). Interestingly, σ^AntA^ expression and antimycin production diminish upon differentiation (Seipke et al., 2014), suggesting that the expression/activity of σ^AntA^ may be “switched off” by some specific regulatory mechanism that is coupled with differentiation, yet needs further investigation.

In summary, ECF σ factors play essential roles in responding to signals and modulate *Streptomyces* metabolism. However, compared to various other ECF σ factors in this particular bacterial species, researches investigations on ECF σ factors and the mechanism underlying their response to signals are limited.

Further investigations on ECF σ factors, the corresponding signals, and the relationship between these signals and biological processes are necessary to enrich our understanding of the *Streptomyces* regulatory network.

## Conclusion

Transcriptional regulation is a crucial mechanism in modulating gene expression, and RNAP is the key element in transcription. Sigma factors are the only variable elements of holo-RNAP, indicating that the selection of a particular target gene is mainly dependent on σ factors. In most cases, unlike other transcriptional factors that upregulate or downregulate gene expression, σ factors act as “switches” that turn expression on or off. In this mini-review, we focused on the members of the σ^70^ family in *Streptomyces*, illustrating their function and underlying mechanisms.

*Streptomyces* species undergo complex morphological differentiation and synthesize various secondary metabolites, which are governed by a complex regulatory network. As a crucial component of the transcriptional regulatory system, σ factors fine-tune gene expression and regulate metabolic fluxes. Thus, engineering of specific σ factors is a feasible method of generating high-producing antibiotic strains (Zhuo et al., 2010), which requires detailed and precise knowledge of σ factor function. However, except for very few members, the biological function of most σ factors and whether these are related to secondary metabolism remains unclear. Thus, further investigations on the functions of *Streptomyces* σ factors should be conducted.

*Streptomyces* has a subtle signal sensing and transduction network that responds to extracellular and intracellular signals. Recent studies have revealed that the majority of antibiotic biosynthesis gene clusters in *Streptomyces* are not expressed, and these clusters are described as “silent.” These silent metabolites are not produced under normal conditions, suggesting that special signals are needed to activate them (reviewed by Okada and Seyedsayamdost, 2017). Sigma factors may respond to some special signals and activate these silent antibiotic synthetic gene clusters, thereby extending our arsenal of bioactive metabolites. Hopefully, future investigations on σ factors may improve our understanding of the *Streptomyces* signal transduction system, thereby promoting antibiotic production and the discovery of novel secondary metabolites.

## Author Contributions

DS, CL, and JZ conducted the literature study and wrote the draft manuscript. WL edited and revised the manuscript.

## Conflict of Interest Statement

The authors declare that the research was conducted in the absence of any commercial or financial relationships that could be construed as a potential conflict of interest.
